# *16p11.2* microdeletion enhances gene expression variability between human IPSC-derived forebrain interneuron progenitor cells in culture

**DOI:** 10.3389/fnmol.2026.1872405

**Published:** 2026-07-08

**Authors:** Yifei Yang, Idoia Quintana Urzainqui, Thomas Pratt

**Affiliations:** 1 Simons Initiative for the Developing Brain, University of Edinburgh, Edinburgh, United Kingdom; 2 Edinburgh Medical School Biomedical Sciences, Institute for Neuroscience and Cardiovascular Research, University of Edinburgh, Edinburgh, United Kingdom; 3 Developmental Biology Unit, European Molecular Biology Laboratory (EMBL), Heidelberg, Germany

**Keywords:** *16p11.2* syndrome, development, gene regulatory network, heterogeneity, induced pluripotent stem cells, neurodevelopmental conditions, regulon, single cell RNA sequencing

## Abstract

The 574-kilobase pair *16p11.2* microdeletion raises a person’s odds for neurodevelopmental and energy balance conditions, particularly autism and obesity, with considerable clinical heterogeneity, and how much this reflects genetic versus environmental or stochastic factors is unclear. GABAergic forebrain interneurons originate from progenitors residing in the ventricular zones of the fetal ventral telencephalon, and their perturbation is implicated in *16p11.2* phenotypes, prompting investigation of how the *16p11.2* microdeletion impacts their development. Here we studied human-induced pluripotent stem cell (IPSC) derived ventral telencephalic interneuron progenitors in two-dimensional culture, comparing IPSCs isogenic except for a heterozygous *16p11.2* microdeletion to minimize confounding effects of genetic background. Single-cell RNA sequencing generated single-cell transcriptome populations for comparative bioinformatics, revealing hundreds of differentially expressed transcripts, many associated with cell signaling, chromatin biology, and neurodevelopmental conditions. Pertinently, we find that transcript level variation is significantly greater between *16p11.2* heterozygous progenitors than their isogenic wild type counterparts both for sets of genes comprising regulons, gene-sets functionally connected by transcription factor regulation, and for randomly selected gene sets. This indicates that the *16p11.2* locus itself has a genome-wide property in stabilizing transcription between cells. Regulons with the greatest increased variability in *16p11.2* heterozygous progenitors exhibit strong enrichment for cell cycle-related genes, and many are regulated by transcription factors themselves associated with autism and/or obesity, suggesting the hypothesis that enhanced transcriptional variation contributes to *16p11.2* microdeletion phenotypes.

## Introduction

Copy number variation (CNV) of the 574-kilobase pair (kbp) *16p11.2* locus in the region BP4-BP5 on the short arm of human chromosome 16 is the genetic risk factor for *16p11.2* syndrome. In humans, the *16p11.2* locus is flanked by 150kbp repeat sequences, and misaligned meiotic homologous recombination generates reciprocal microdeletion and microduplication *16p11.2* alleles, allowing for *16p11.2* CNVs to arise *de novo* at relatively high frequency (D’Angelo et al., 2016; Nuttle et al., 2016). The *16p11.2* locus is a dosage sensor with deviation from a dosage of two *16p11.2* alleles, heterozygosity for the *16p11.2* microdeletion or microduplication, each associating with *16p11.2* neuropsychiatric, anatomical, and energy balance phenotypes (Jacquemont et al., 2011; Maillard et al., 2016; Weiss et al., 2008). Harboring the *16p11.2* microdeletion increases the odds (odds ratio (OR)) of developing obesity (BMI > 30 kg/m^2^) 43-fold, autism 40-fold, any psychiatric disorder 9-fold, and ADHD 4-fold (Niarchou et al., 2019; Walters et al., 2010). Most *16p11.2* microdeletion carriers present with more than one symptom, with overall ~70% being obese and/or 15–25% exhibiting autism, epilepsy, intellectual disability, and/or macrocephaly (Chung et al., 2021; Rein and Yan, 2020). The variable penetrance and expressivity of the *16p11.2* microdeletion between different carrier individuals have hitherto been attributed to genetic variation outside the *16p11.2* locus and environmental factors, including the maternal prenatal environment (Girirajan et al., 2012; Hudac et al., 2020). In non-*16p.11.2* contexts the odds of developing obesity associated with monogenic risk factors are modified by polygenic risk (Akbari et al., 2021). Overall, it remains an open question if the *16p11.2* locus itself contributes to clinical variation.

Studies using human and animal models have identified *16p11.2* neuronal phenotypes at various stages of development, including differential gene expression, cell proliferation, signaling, cell and tissue anatomy, electrophysiology, and behaviour (Antoine et al., 2019; Arbogast et al., 2016; Blumenthal et al., 2014; Connacher et al., 2022; Deshpande et al., 2017; Fetit et al., 2023; Horev et al., 2011; Migliavacca et al., 2015; Pucilowska et al., 2018, 2015; Qiu et al., 2019; Qureshi et al., 2014; Roth et al., 2020; Sundberg et al., 2021; Urresti et al., 2021; Yang et al., 2023). Reduced dosage of individual *16p11.2* genes, including *ALDOA*, *KCTD13*, *MAPK3*, *TAOK2, and MVP,* has been reported to impact the proliferation and/or differentiation of neural progenitors (Blaker-Lee et al., 2012; de Anda et al., 2012; Escamilla et al., 2017; Golzio et al., 2012; Kizner et al., 2020; Pucilowska et al., 2012; Richter et al., 2019; Yadav et al., 2017). How the *16p11.2* locus acts and the extent to which simultaneously reduced dosage of *16p11.2* genes synergize to produce microdeletion phenotypes are not fully understood.

GABAergic inhibitory neurons (INs or interneurons) form functional circuits with excitatory neurons in the cerebral cortex and subcortically, and shifting the balance between excitation and inhibition has long been hypothesized to contribute to autism and its co-occurring conditions (Antoine et al., 2019; Bozzi et al., 2018; Nelson and Valakh, 2015; Puts et al., 2017; Rapanelli et al., 2017; Robertson et al., 2016; Rubenstein and Merzenich, 2003). Cortical IN progenitors reside in the ventricular zones of the ventral telencephalon primarily in the medial ganglionic eminence (MGE) and caudal ganglionic eminence (CGE) where they express combinations of transcription factors including *NKX2.1*, *OLIG2*, *ASCL1*, *HES1*, *SOX2*, *NR2F2*, and *PAX6* depending on their position and subsequently differentiate, express transcription factors including *DLX2* and *LHX6* and GABAergic markers including *SST* (Somatostatin) and *SLC32A1* (VGAT) before migrating to their target areas (Hansen et al., 2013; Lim et al., 2018; Ma et al., 2013; Marin, 2025). In developing human embryos, many genes in the *16p11.2* locus are expressed in IN progenitors, suggesting they may be vulnerable to altered *16p11.2* dosage (Morson et al., 2021; Shi et al., 2021; Yu et al., 2021). Consistent with this hypothesis, we recently showed that isogenic human IPSC-derived IN progenitors in *16p11.2^+/-^* organoids exhibit an extended cell-cycle and accelerated maturation and, intriguingly, increased inter-organoid variability in these and other phenotypes despite being genetically homogenous, suggesting that the *16p11.2* locus itself regulates variability in this context (Fetit et al., 2023).

Identifying genes with significantly different average expression levels between genotypes has provided valuable insights into genetic mechanisms. By its nature, this approach tends to ignore the dimension of gene expression variability, and this has hitherto been far less explored. Here, we use comparative single cell transcriptomics of human IPSC-derived IN progenitor populations to gain insight into the molecular effects of the *16p11.2* microdeletion on both average gene expression levels and gene expression variability between cells. To minimize the confounding effects of variable genetic background on the *16p11.2* phenotype, we derive the IN progenitors from IPSCs isogenic except for whether or not they are heterozygous for the *16p11.2* microdeletion (Fetit et al., 2020; Liu et al., 2013; Tai et al., 2016). We first focus our analysis on transcripts whose average expression levels are significantly different and then concentrate on how global transcript expression level variability between cells is affected by the *16p11.2* microdeletion. We find hundreds of genes are differentially expressed at relatively modest levels in *16p11.2^+/-^* IN progenitors many of which are involved in chromatin biology and cell signaling. Our major finding is a general increase in cell–cell variability in gene expression between isogenic *16p11.2^+/-^* IN progenitor cells compared to their wild-type counterparts. We conclude the *16p11.2* locus has a global stabilizing effect on gene expression, including genes functionally associated with the cell cycle, brain development, and *16p11.2* symptoms.

## Methods

### DNA SNP microarray analysis

Illumina HumanCytoSNP-12 v2.1 array analysis was used to assess genomic structure in the three deletion (DELD5, DELH7, and DELC5) and the two control (FACS52 and FACS53) human IPSC lines used for this study, and their ancestral GM8 line.

### *In vitro* differentiation of human IPSC lines into INs

The human IPSC lines used for this study were CRISPR/Cas9 engineered from the parental GM8 IPSC line to recapitulate the genomic architecture of the *16p11.2* microdeletion in patients (*16p11.2^+/–^*) or isogenic controls derived from the same GM8 parental line without CRISPR/Cas9 engineering (*16p11.2^+/+^*) (Tai et al., 2016). IPSCs were differentiated along a ventral telencephalic IN trajectory in six independently grown cultures, three of each genotype. Three *16p11.2^+/–^* IPSC lines, each produced by a different targeting event (DELD5, DELC5, and DELH7), were each used to generate a separate culture, while one isogenic *16p11.2^+/+^* control line (FACS5.2) was used to generate one culture, and another (FACS5.3) was used to generate two cultures. We refer to *CON* (*16p11.2^+/+^*) cultures as C1, C2, and C3 and the *DEL* (*16p11.2^+/–^*) cultures as D1, D2, and D3: C1 = FACS5.2; C2 = FACS5.3; C3 = FACS5.3; D1 = DELD5; D2 = DELC5, and D3 = DELH7. Differentiation of IPSCs was performed in 2-dimensional (2D) culture as described by Liu et al. (2013) (Supplementary Figure S2A). For each sample, IPSCs were grown in maintenance medium composed of a 1:1 mix of mTeSR™1 (Stem Cell Technologies) and Essential 8 TM Medium (Thermo Fisher Scientific). On day 0, IPSCs were lifted and plated in non-adherent plates to allow the formation of embryoid bodies (EBs). On day 4, pluripotency media were replaced by Neural Induction Media (NIM). On day 7, EBs were plated in Matrigel-coated plates (Matrigel Matrix, Corning) and allowed to form rosette structures. On day 10, the Shh agonist purmorphamine (Stem Gent, 1.5 μM) was added to the media to induce GABAergic differentiation. On day 16, rosettes were manually selected, lifted with a 10 μl pipette tip, and transferred to a non-adherent plate to allow the formation of neurospheres. B27 supplement (1:50, without Vitamin A, Gibco) was added to the media. On day 26, neurospheres were incubated in Accutase solution for 5 min at 37 °C and dissociated. Single cells were plated in 24-well plates coated with PLO (Poly-L-ornithine, Sigma-Aldrich) and laminin at a density of 250,000 cells per well in Neuronal Differentiation medium containing 1 mM cAMP and 100 μg/mL of BDNF, GDNF, and IGF1. Cultures were fed every 3 days and left to mature until day 80.

### Immunofluorescence

Cultures were fixed in 4% paraformaldehyde (PFA) for 30 min to 2 h, followed by permeabilization in 0.1% Triton X-100 diluted in PBS (PBST) and blocked in 10% donkey or goat serum in PBST for 30 min at room temperature. Then incubated overnight at 4 °C with primary antibodies, and PBST was used to wash off the primary antibodies, followed by incubation with fluorescent-conjugated secondary antibodies in blocking solution for 1 h. Finally, nuclei were visualized with DAPI (Hoechst 33342, BD Pharmingen 561,908, 5 μg/mL) and mounted for microscopy under coverslips using Vectashield Hardset (Vector Labs) for imaging on an epifluorescence microscope. Primary antibodies: FOXG1 (Rabbit, Abcam ab18259, diluted 1/100) and TUJ1 (Mouse, Biolegend 801,203, diluted 5 μL/ million cells) in blocking solution. Secondary antibodies: Anti-Rabbit-488 (Goat, Alexa Fluor 488, Abcam ab150077, dilution 1/200) and Anti-mouse-647 (Goat, Alexa Fluor 647, Abcam ab150115, 1/200).

### cDNA library preparation and single cell RNA sequencing

Cultures were dissociated into a single cell suspension using Accutase. Cells were checked for viability by Trypan Blue staining and cell density estimated in an automated cell counter (Countess, ThermoFisher). Multiplex i7 Barcoded cDNA libraries were prepared for single-cell RNA sequencing using 10X Chromium Next GEM v3.1 Single Cell 3’ Library and Gel Bead kit and loaded onto the 10x Chromium instrument through a Next GEM v3.1 Chip G. Samples were loaded into independent channels at a density of 10^6^ cells/ml. Libraries were constructed following the manufacturer’s protocol. Bioanalyzer traces were assessed for RNA quality, and all samples were run on one lane on a NovaSeq S1 at 750 M read pairs. Multiplex i7 Barcoded cDNA libraries were prepared for single cell RNA sequencing using 10X Chromium Next GEM v3.1 Single Cell 3’ Library and Gel Bead kit and loaded onto a Next GEM v3.1 Chip G for 10x sequencing.

### Single-cell RNA-Seq data processing and clustering

CellRanger (ver. 6.0) was used to map fastq files to the human genome assembly version GRCh38. Raw count matrix was processed and analyzed using the *Seurat* R package (ver. 4.3.0). Quality control was performed to filter low-quality cells. Cells were removed if they met any of the following criteria: (1) gene numbers less than 800, (2) gene numbers greater than 10,000, (3) UMI greater than 200,000, (4) percentage of mitochondrial RNA UMIs greater than 10%, (5) percentage of ribosome RNA UMIs greater than 40%, and (6) percentage of red cell genes (“HBA1,” “HBA2,” “HBB,” “HBD,” “HBE1,” “HBG1,” “HBG2,” “HBM,” “HBQ1,” and “HBZ”) greater than 0.1%. Following quality control, data were normalized using the *NormaliseData* function, and top 2000 highly variable features (HVGs) were identified using the *FindVariableFeatures* method. Dimensionality reduction was performed via principal component analysis (PCA), followed by non-linear dimensionality reduction using uniform manifold approximation and projection (UMAP) for visualization. Graph-based clustering was performed using the *FindNeighbors* and *FindClusters* functions with the resolution 0.8. Elimination of batch effects between samples was achieved by Harmony (ver. 0.1.0).

### Cell type annotation

Cell types were annotated based on the expression of canonical marker genes. To validate these annotations, a heatmap of the top marker genes for each cluster was generated using the *ggplot* R package (ver. 3.4.4). Clusters were assigned to specific cell types by comparing marker expression profiles against known cell-type signatures. Transcriptomic correlation between single cell transcriptomes of *in vitro* differentiated IPSC cell types (current study) and single cell transcriptomes of *in vivo* annotated cell types from gestational week 9–12 foetal human ganglionic eminences (Yu study), where P1–P6 are IN progenitors and LGE, MGE, and CGE are more differentiated IN precursor cells (Yu et al., 2021). Pearson correlation coefficients were calculated across the intersecting highly variable genes between the datasets.

### Differential expression analysis via pseudo-bulk aggregation

To identify robust differentially expressed genes (DEGs) based on average expression between conditions, we performed pseudo-bulk analysis. Single-cell gene expression counts were aggregated to the sample level by summing the counts for each gene across all cells within the same biological replicate and cell type. This generated a “pseudo-bulk” count matrix, which was then used as input for *DESeq2* (ver. 1.34.0). Differential expression testing was performed using a standard likelihood ratio test within the DESeq2 framework. Individual transcripts with an adjusted *p*-value < 0.05 were considered statistically significant DEGs.

### Biological function of DEGs

To investigate the biological functions of the DEGs, gene ontology (GO) annotation and pathway enrichment analysis of the DEGs were carried out using the Metascape web tool^1^ (Zhou et al., 2019) with the ontology sources of GO Biological Processes, KEGG Pathway, Reactome, Gene Sets, and WikiPathways Pathway. Genes expressed in >5% of cells were used as background. Disease enrichment analysis was performed using the DisGeNET platform. The list of differentially expressed genes was tested for enrichment against known disease-associated gene sets. Statistical significance was assessed using a hypergeometric test, with Benjamini–Hochberg correction for multiple testing (adjusted *p*-value < 0.05). Gene-set enrichment analysis (GSEA) was performed to identify enrichment of terms associated with biological processes (Subramanian et al., 2005).

### Genetic risk for autism (ASD) and obesity (BMI)

To evaluate the disease risk associated with dysregulation of specific genes, we integrated differential expression data with genetic association metrics for body mass index (BMI) and Autism spectrum disorder (ASD). BMI-associated risk single nucleotide polymorphisms (SNPs) were acquired from the UK Biobank genome-wide association study (GWAS) summary statistics (Sudlow et al., 2015). Variants were mapped to genes within a 33.5 kb flanking window to extract corresponding effect sizes BETA (*β*) (Reynolds, 2022; Available at: https://doi.org/10.5281/zenodo.6127446), and BETA values were then averaged for each gene. ASD risk genes and their associated metrics were sourced from previously published large-scale exome sequencing data (Rolland et al., 2023). To visualize the intersection of genetic risk and fold change (FC) transcriptomic dysregulation, BETA values were plotted against log_2_FC values for both BMI and ASD datasets to highlight significant targets.

Transcriptomic downregulation can further be translated into a functional genetic risk framework by approximating an effective loss-of-function (LOF) allele dosage from differential expression fold-change (FC). In a standard genetic model, risk is defined as the product of the effect size and the variant dosage: *Risk = BETA × Dosage* (Collister et al., 2022). We modelled the RNA expression FC—calculated from DESeq2 log_2_FC results comparing deletion (*DEL*) to control (*CON*) samples—as a proxy for LOF dosage. Under the assumption that normal expression (FC = 1.0) is equivalent to a wild-type state (Dosage = 0), a 50% reduction (FC = 0.5) mimics a heterozygous LOF state (Dosage = 1), and complete absence of expression (FC = 0) mimics a homozygous LOF knockout (Dosage = 2), allowing the effective dosage for each downregulated transcript to be calculated as *Dosage = 2 - (2 × FC)*. The risk associated with each individual transcript downregulated in DEL samples can be calculated as *Risk = BETA × Dosage*. A comparative transcriptomic proxy of polygenic risk is calculated by summing all the individual transcript risk values associated with a particular condition (in this case, ASD or BMI), analogous to calculating polygenic risk by summing risk associated with individual alleles in a genotype (Collister et al., 2022). To focus on downregulation of transcripts more likely to model a hypomorphic allele, the polygenic risk calculation only included transcripts downregulated to less than 70% of *CON* levels (FC < 0.7) in *DEL* samples. This method calculates a polygenic risk score for the *DEL* genotype compared to the *CON* genotype. Each transcript in *CON* samples has an FC = 1 compared to itself, a Dosage = 0, and a Risk = 0 by definition. Therefore, a positive polygenic risk score value indicates increased overall risk caused by the *16p11.2* microdeletion in *DEL* samples, while a negative value indicates a protective effect. We did not apply this approach to transcripts upregulated in *DEL* because overexpression does not predictably correspond to hypomorphic alleles.

### Single-cell regulatory network analysis

Gene regulatory networks (regulons) were inferred using *SCENIC* (Single-Cell regulatory Network Inference and Clustering) (ver. 1.3.1) (Aibar et al., 2017). Co-expression modules were identified using the *GRNBoost2* function, and false positives were filtered based on cis-regulatory motif analyses (cisTarget). The regulons were identified in the whole *CON* and *DEL* populations separately. Neurodevelopmentally regulated target genes identified by Chu et al. (2021) were included for each regulon.

### Expression variation analysis (EVA)

To quantify the differential heterogeneity of gene regulation across conditions independently of changes in mean expression, we applied Expression Variation Analysis (EVA) (Davis-Marcisak et al., 2019). The mathematical principle of EVA relies on a non-parametric, distance-based test for multivariate dispersion. For a given gene set (e.g., a SCENIC regulon or random gene set), EVA calculates a cell-to-cell dissimilarity matrix based on the transcriptional profiles of those specific genes. It then quantifies the multivariate dispersion—or average distance—between cells within each biological condition (a sample cell population). The primary biological assumption of this approach is that regulatory destabilization manifests as increased transcriptional variance, causing cells of the same phenotype to drift further apart in their expression states. A higher EVA score indicates greater multivariate dispersion, signifying increased transcriptional heterogeneity for that regulon gene set. We applied EVA to assess heterogeneity in two types of gene sets. One was regulons derived from SCENIC, and another one was random gene sets, generated by the online tool MOLBIOTOOLS^2^ with default setting. Any gene that was expressed in less than 5% of all cells was excluded from further EVA.

## Results

*16p11.2^+/-^* and *16p11.2^+/+^* genotypes are subsequently referred to as *DEL* (heterozygous for *16p11.2* microdeletion) and *CON* (control), respectively.

### Genomic structure of IPSC lines

The human IPSC lines used for this study were subjected to single-nucleotide polymorphism (SNP) analysis (Supplementary Figure S1). This confirmed that the IPSC cells were male (XY) and heterozygous for a deletion spanning the *16p11.2* locus from *SPN* to *CORO1A* in the *DEL* lines (orange shading) and not in control *CON* lines, as previously reported (Tai et al., 2016). The most frequent genomic feature identified by SNP analysis was loss of heterozygosity (green shading) present in the ancestral GM8 line and preserved *CON* and *DEL* lines. Outside the *16p11.2* locus, there were a small number of CNVs, most of which had emerged after divergence from GM8. =There were a few homozygous deletions (red shading) and homozygous duplications (purple shading); however, most did not span genes, so they are not expected to affect gene dosage. In conclusion, the only genomic change consistently distinguishing the *CON* and *DEL* lines used in this study is the *16p11.2* microdeletion itself, confirming these lines as overwhelmingly isogenic and suitable for investigating the molecular consequences of the *16p11.2* microdeletion without genetic variation outside the *16p11.2* locus as a confounding factor.

### Characterization of IPSC-derived cells

**Figure 1:**
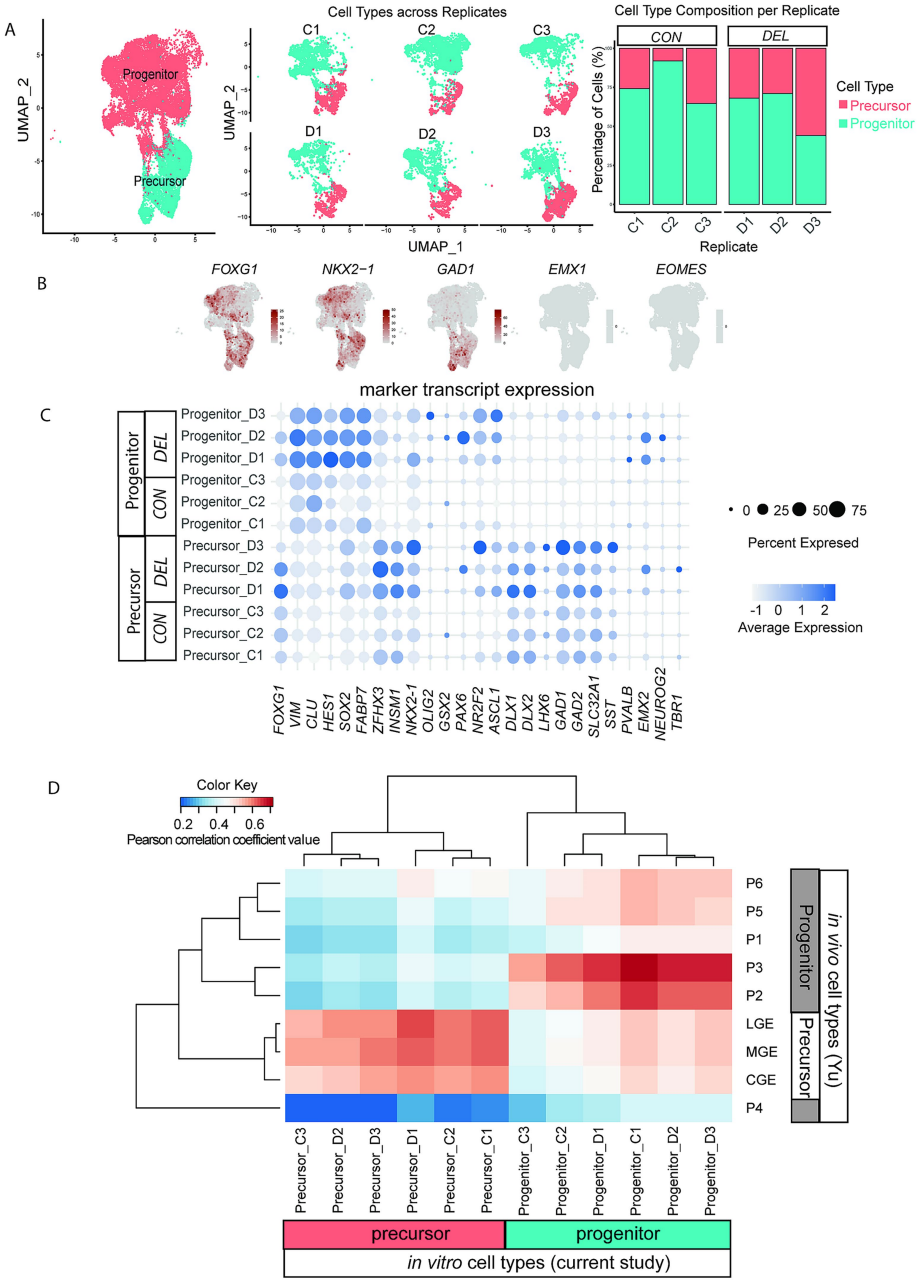
**(A)** (left panel) UMAP showing dimensional reduction of the single cell transcriptomes for all samples of CON and DEL cells, each dot representing a single cell transcriptome, resolves into two main clusters identified as ‘progenitors’ and ‘precursors’ (middle panel) shows UMAP for each of the CON (C1, C2, and C3) and DEL (D1, D2, and D3) samples with (right panel) showing the composition of progenitors and precursors in each sample. **(B,C)** Marker transcript expression **(B)** Gradient plots shows expression of transcripts mapped onto the UMAP shown in **(A)**: The telencephalic marker FOXG1 and ventral telencephalic GABAergic markers NKX2.1 and GAD1 are expressed while dorsal telencephalic markers EMX1 and EOMES exhibit no detectable expression **(C)** Dot plot showing marker transcript expression in CON and DEL progenitor and precursor populations from all samples individually, for each transcript the dot indicates percent of expressing cells and average expression (see key) across cell populations. Markers: ventral and dorsal telencephalon (FOXG1); neural progenitor (VIM, CLU, HES1, SOX2, and FABP7) and early post-mitotic neuron (ZFHX3 and INSM1) respectively; MGE progenitor (NKX2.1, OLIG2, and GSX2), CGE and dorsal telencephalon progenitor (PAX6 and NR2F2); GABAergic post-mitotic neuron (DLX1, DLX2, GAD1, GAD2, SLC32A1, LHX6, SST, and PVALB); and dorsal telencephalic (EMX2, NEUROG2, and TBR1). **(C)** Transcriptomic correlation between in vitro differentiated individual cell types (‘current study’, columns) and cell types from foetal human ganglionic eminences *in vivo* (‘Yu study’, rows), where P1–P6 are IN progenitors and LGE, MGE, and CGE are more differentiated IN precursor cells. Similarity between cell types was determined by calculating the Pearson correlation coefficient of average gene expression profiles using the intersecting highly variable genes between both datasets. The heatmap shading and accompanying scale bar indicate the strength of the Pearson correlation coefficient, ranging from the strongest positive correlation (deep red) to the weakest/negative correlation (deep blue). Hierarchical clustering of rows and columns showing transcriptomic similarity above and to the left of the heat-map.

Human IPSC cells were differentiated for 80 days following the protocol of Liu et al. (2013) summarized in Supplementary Figure S2A and checked for the telencephalic transcription factor FOXG1 and the post-mitotic cytoplasmic neuronal marker TUJ1 proteins. The vast majority of *CON* and *DEL* cell nuclei were FOXG1^+^ (Supplementary Figures S2B,D) and many had acquired a TUJ1^+^ post-mitotic neuronal fate (Supplementary Figures S2C,E), confirming telencephalic neuronal identity. Single-cell RNA sequencing of three *CON* (C1, C2, and C3) and three *DEL* (D1, D2, and D3) cultures yielded 33,954 cells across the six samples, of which 14,916 comprised empty droplets, doublets, and cells with high levels of mitochondrial or ribosomal reads that were removed, leaving 19,038 high-quality cell transcriptomes for further analysis. We clustered cells using UMAP, revealing two distinct populations (Figure 1A).

The larger population (12,713 cells) comprised cells expressing telencephalic marker *FOXG1*, neural progenitor markers (*VIM*, *CLU*, *HES1, SOX2, and FABP7*), and markers expressed by ventral telencephalic LGE, MGE, or CGE IN progenitors (*NKX2.1*, *OLIG2*, *GSX2*, *PAX6*, *NR2F2*, and *ASCL1*), indicating a forebrain IN progenitor fate (Figures 1B,C). In contrast, there was low or undetectable expression of key dorsal telencephalic progenitor markers (*EMX1* or *EOMES* (*TBR2*)), arguing against dorsal telencephalic progenitor fate (Figure 1B). The proportions of expressing cells are likely underestimated due to ‘zero inflation’, a technical property where scRNAseq sometimes returns a false negative zero value for a transcript, but it is unlikely that this explains the complete absence of *EMX1* or *EOMES* expression (Figure 1B). Taken together, this suggests most of our IPSC-derived cells closely model the ventral telencephalic IN progenitor state. The smaller cluster (6,235 cells) shows upregulation of markers characteristic of early differentiating post-mitotic neural cells (*ZFHX3* and *INSM1*) and associated with IN status (*GAD1*, *GAD2*, *LHX6*, *SLC32A1* (*VGAT*), *SST* (Somatostatin)), and there is low expression of *PAVLB* (Parvalbumin), which is expressed later in development (Figures 1B,C).

We next sought to gain a more global mapping of our *in vitro* IPSC-derived single-cell transcriptome to the IN developmental trajectory *in vivo*. A recent study categorised single cell transcriptomes obtained from GW 9–12 human foetal ganglionic eminences as IN progenitors (denoted P1, P2, P3, P4, P5, and P6) and more differentiated IN precursors (denoted LGE, MGE, and CGE) (Yu et al., 2021). Comparison between our *in vitro* cell types and these *in vivo* cell types showed that the clusters we had identified as *in vitro* IN progenitors strongly correlated with the *in vivo* IN progenitors classed as P2 and P3 (the strongest red shading in Figure 1D indicating the highest Pearson correlation coefficient) (Shi et al., 2021; Yu et al., 2021). The remainder of the *in vitro* cell population correlated with more differentiated LGE, CGE, and MGE precursor cell classes in the Yu study (Figure 1D).

Overall, both analysis of individual marker transcripts (Figures 1B,C) and global transcriptomic correlation to *in vivo* cell-types (Figure 1D) strongly indicate the cultures primarily comprising a meaningful correlate of human ventral telencephalic GABAergic IN progenitors and their IN precursor descendants.

### Differential gene expression between CON and DEL progenitor populations

**Figure 2:**
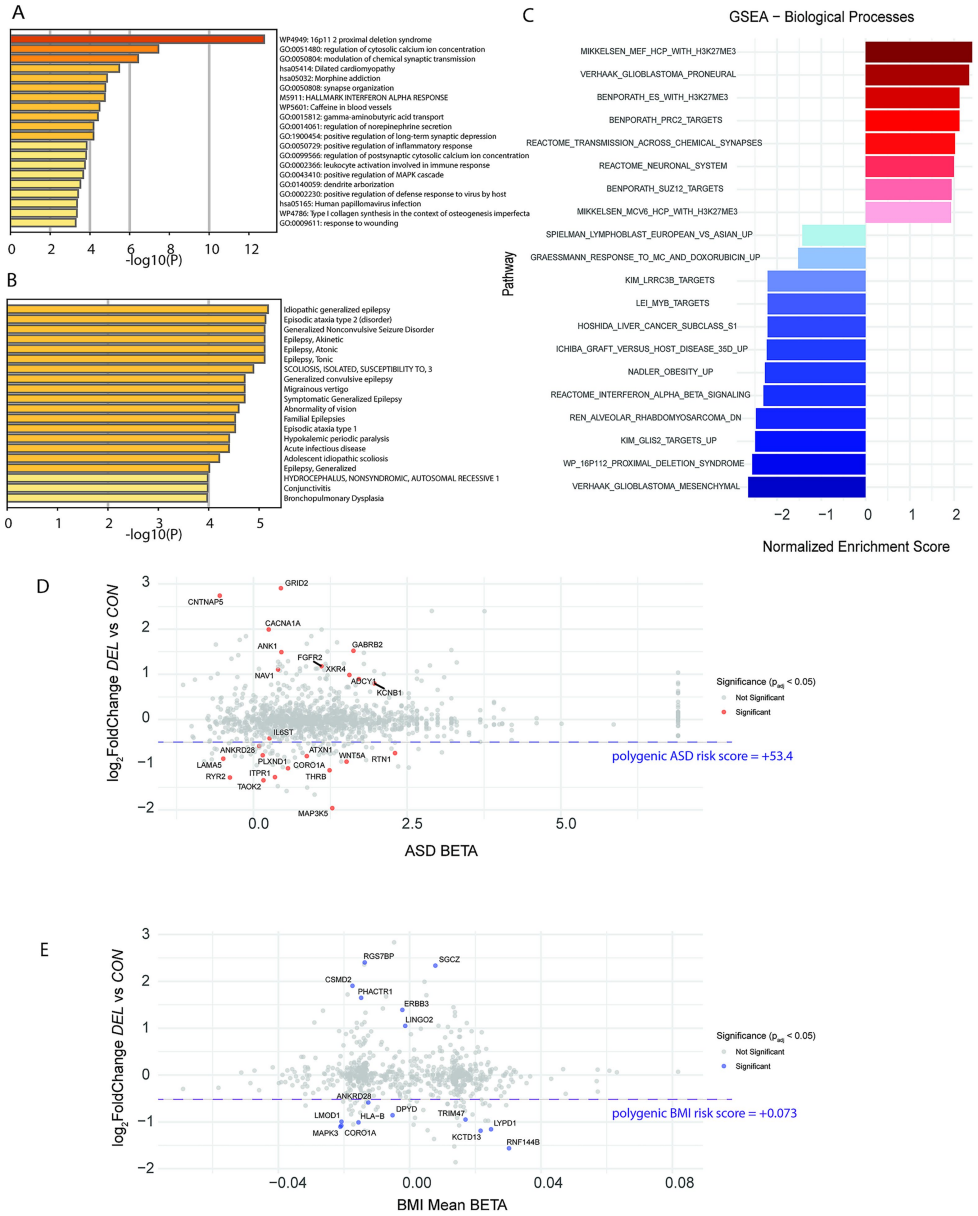
Analysis of transcripts differentially expressed between CON and DEL progenitor cells. **(A,B)** Enrichment of transcripts significantly differentially expressed (DESeq2 padj<0.05) between CON and DEL progenitors in **(A)** Gene ontology (GO) or **(B)** Disease (DisGeNET) terms. **(C)** Gene set enrichment analysis (GSEA) of all transcripts, where positive and negative normalized enrichment scores indicate enrichment of genes in a given gene-set in upregulated or downregulated transcripts, respectively. **(D,E)** Plots of CON vs. DEL Log¬2Fold-Change (Log2FC) transcript expression versus genetic risk (BETA value) for **(D)** ASD and **(E)** BMI. The polygenic risk score (see Methods) for **(D)** ASD or **(E)** BMI was calculated by summing individual risks associated with each transcript downregulated in DEL samples to less than 70% of CON levels (Log¬2FC < -0.5 indicated by dashed horizontal lines). Individual transcripts significantly differentially expressed (DESeq2 padj<0.05) between CON and DEL progenitors are colored in and named.

We next used DeSEq2 to identify differences in average ‘pseudo-bulk’ gene expression between *CON* and *DEL* progenitors. After filtering out genes expressed in less than 5% of cells, there were 250 significantly (DeSeq2 *p*_adj_ < 0.05) differentially expressed genes (DEGs), 104 upregulated and 146 downregulated in *DEL* progenitors, respectively (Supplementary Table S1). Gene ontology (GO) analysis using the significant DEGs as input revealed that the most enriched term was *16p11.2* proximal deletion syndrome (Figure 2A), consistent with downregulation of *16p11.2* transcripts in *DEL* cells, as has been observed in other studies. There was also enrichment for other terms relevant to brain development, including calcium signaling, the synapse, and signaling pathways. DisGeNET analysis using the significant DEGs as input showed enrichment of the DEGs in a number of human disease states with terms related to epilepsy and seizures, prominent features of *16p11.2* syndrome, being the most pronounced (Figure 2B).

Gene set enrichment analysis (GSEA) ranks transcripts by their fold-change regardless of whether they are individually called as statistically significant DEGs and identifies gene-sets which are either enriched in upregulated transcripts (positive normalized enrichment score) or in downregulated transcripts (negative normalized enrichment score) (Subramanian et al., 2005). In progenitor cells, the majority of top terms with the highest positive enrichment were gene-sets linked to chromatin biology (MIKKELSEN_MEF_HCP_WITH_H3K27ME3, BENPORATH_ES_WITH_H3K27ME3, BENPORATH_PRC2_TARGETS, and BENPORATH_SUZ12_TARGETS, MIKKELSEN_MCV6_HCP_WITH_H3K27ME3), cancer-related terms were present in both positively (VERHAAK_GLIOBLASTOMA_PRONEURAL) and negatively (HOSHIDA_LIVER_CANCER_SUBCLASS_S1 and VERHAAK_GLIOBLASTOMA_MESENCHYMAL) enriched. The *16p11.2* locus transcripts (WP_16P112_PROXIMAL_DELETION_SYNDROME) were negatively enriched and, interestingly, so was an obesity related term (NADLER_OBESITY_UP) (Figure 2C).

The *16p11.2* microdeletion increases the odds of developing autism and obesity (body mass index (BMI) > 30 kg/m^2^) the most in *16p11.2* microdeletion carriers; however, unlike epilepsy, neither feature prominently in our DisGeNET results (Figure 2B). We took advantage of publicly available genome-wide association studies (GWAS) data reporting the association between single nucleotide polymorphism (SNP) loci and either autism as a binary trait (Rolland et al., 2023) or body mass index (BMI) as a continuous trait from UK BIOBANK (Bycroft et al., 2018). While a SNP associated with altered odds of developing a trait is itself unlikely to be directly causing the condition, it may be genetically linked by being in close proximity to the causal allele in the genomic DNA, making mutations in genes near the SNP strong candidates. For GWAS studies, the ‘effect size’ or *β* (BETA), calculated from the odds ratio (OR) with BETA = log_e_OR, indicates the contribution of each allele to the condition. Increasingly positive BETA indicating increased risk, BETA = 0 indicating no risk, while negative BETA values indicate a risk-reducing (protective) effect (Collister et al., 2022). Plotting the Log_2_Fold-change value against the BETA value for autism (Supplementary Table S2) or obesity (Supplementary Table S3) for each transcript reveals hundreds of transcripts upregulated or downregulated in *DEL* IN progenitors that correspond to genes associated with either ASD (Figure 2D) or BMI (Figure 2E) risk. Polygenic risk scores calculated by summing the individual risks associated with each allele across the genome allow comparison of the overall risk associated with genotypes (Collister et al., 2022). We calculated ASD or BMI genetic risk, based on the assumption that downregulation of a given transcript in *DEL* cell models a hypomorphic genotype, for downregulated transcripts and summed them to give a polygenic risk score for the *DEL* transcriptome (see Methods). While these absolute polygenic risk score values cannot be meaningfully compared between different phenotypes, each gives a quantitative relative measure of the risk associated with *DEL* compared to *CON,* where positive values indicate an overall increased polygenic risk associated with the *16p11.2* microdeletion. Both ASD and BMI return positive values of +53.4 (Figure 2D) and +0.073 (Figure 2E), respectively, indicating an increased risk imparted by downregulation of multiple transcripts.

### Regulon gene-sets exhibit greater variability in DEL progenitors with increased enrichment of genes linked to the cell cycle and cancer

**Figure 3:**
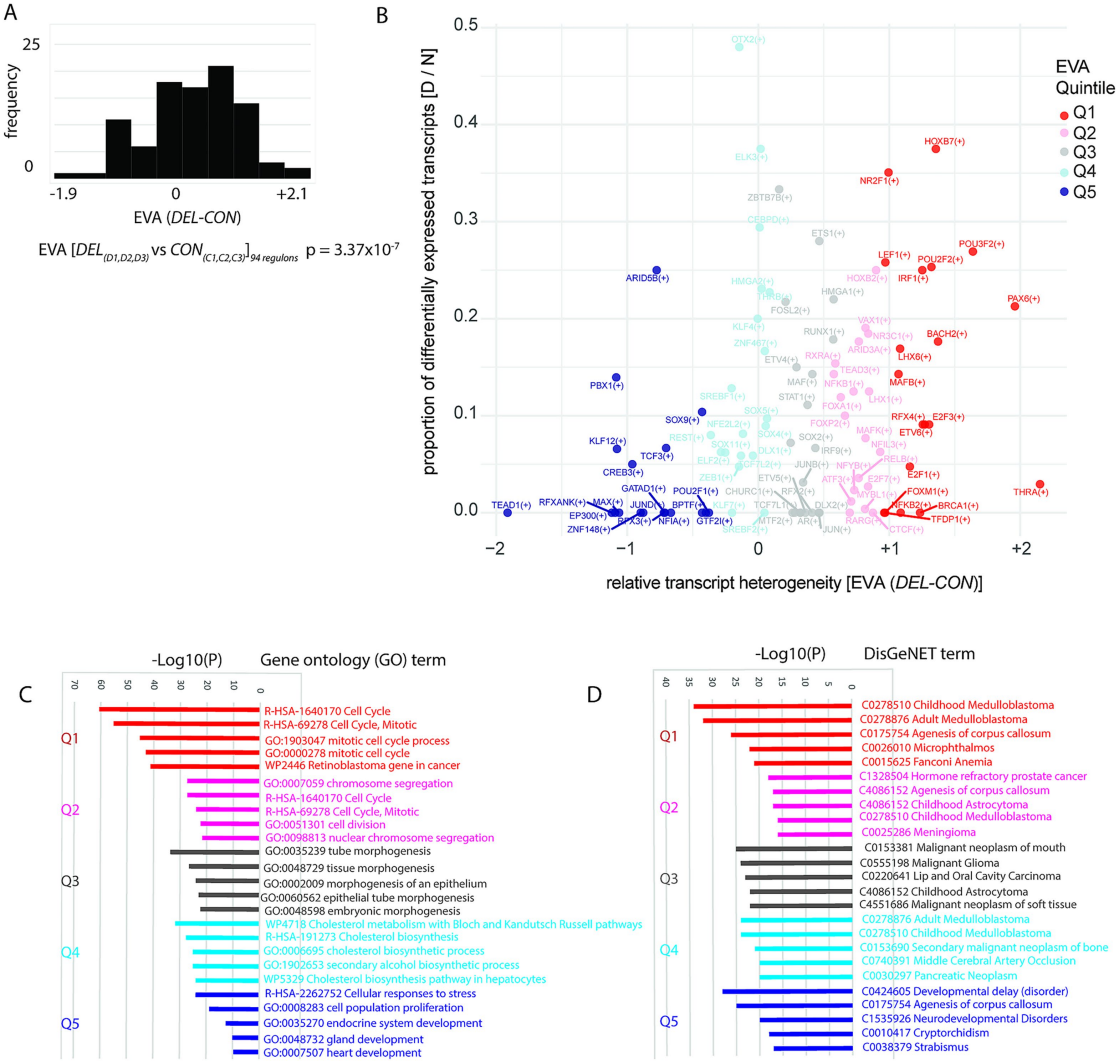
**(A,B)** Comparative expression variation analysis (EVA) of 94 regulon gene sets between CON and DEL progenitor cell populations. EVA(DEL-CON) is the difference between the average EVA scores of DEL and CON progenitor samples, with a positive score indicating increased gene expression variability in DEL samples. **(A)** The distribution of EVA(DEL-CON) values shows a bias to positive scores. Paired t-test comparing EVA values for each regulon between CON and DEL samples (*n* = 94 regulons, 3 samples per genotype) *p* = 3.37×10-7. **(B)** Relationship between the EVA(DEL-CON) value for each regulon and the proportion of target transcripts in that regulon upregulated or downregulated more than 2-fold (D/N). The hubTF for each regulon is indicated (e.g., THRA(+) is the regulon with THRA as the HubTF). Q1-Q5 indicate the quintiles used for gene ontology and DisGeNET, with Q1 comprising the regulons with the greatest increased relative variability in DEL samples. **(C)** Gene ontology (GO) and **(D)** DisGeNET analysis of the combined ‘target’ genes of the regulons in each of the quintiles. In **(C,D)**, the top 5 enriched terms for each quintile and their -log10P values are indicated.

Regulons describe sets of genes functionally connected by transcriptional regulation. A regulon comprises a transcription factor (the HubTF) and the genes it transcriptionally regulates (the targets). SCENIC (Aibar et al., 2017) identified 94 regulons in *DEL* (Supplementary Table S4) and *CON* (Supplementary Table S5) and Expression Variation Analysis (EVA) (Davis-Marcisak et al., 2019) was then used to calculate the transcript level variation (EVA scores) for each of the 94 regulons for all six independent single cell transcriptome populations (C1, C2, and C3 for *CON* and D1, D2, and D3 for *DEL*) (Supplementary Tables S6, S7). A single omnibus paired t-test across all regulons, paired by regulon, comparing the EVA scores in *DEL* vs. *CON* samples revealed a significant global increase in transcriptional variability (*n* = 94 regulons, 3 samples of each genotype, paired t-test *p* = 3.37 × 10^-7^), indicating that the *16p11.2* microdeletion genotype inherently destabilizes gene expression across the regulons examined. Averaging EVA values for *DEL* and *CON* samples separately and calculating the difference gives the EVA(*DEL-CON*) value, where positive values indicate greater gene expression variation in *DEL* samples on average. The distribution of EVA(*DEL-CON*) values shows a clear bias toward positive values (Figure 3A), indicating global destabilization of gene expression in *DEL* compared to *CON* cell populations. Plotting gene expression variability between *CON* and *DEL* samples (EVA(*DEL*-*CON*)) against the proportion of differentially expressed transcripts in each regulon (D/N) reveals a weak positive correlation (correlation coefficient = +0.25) between D/N and EVA(*DEL-CON*) (Figure 3B), indicating that transcript level variability and changes in average expression level are reasonably independent.

The 94 regulons were ranked by EVA(*DEL-CON*) value and divided equally into quintiles Q1 – Q5 (indicated on Figure 3B). To investigate the functional consequences of gene expression variability, all the target genes in each quintile were combined and subjected to GO (Figure 3C) and DisGeNET (Figure 3D) analysis. GO terms related to cell division (‘cell cycle’, ‘DNA replication’, ‘regulation of cell cycle phase’, ‘S-phase’, cell population proliferation’, mitotic cell cycle process’, ‘chromosome segregation’) were represented across all quintiles with enrichment particularly pronounced (highest -log10(P) value indicating higher proportion of genes from a GO term present in the quintile gene-set) in Q1, the quintile containing regulons with the greatest increased variability in *DEL* progenitors, with a trend toward lower significance in quintiles containing successively less variable regulons. This suggests that regulons exhibiting the greatest gene expression variation in *DEL* progenitor cells compared to *CON* cells are functionally involved in cell division. DisGeNET terms associated with cancers (‘Childhood medulloblastoma’, ‘adult medulloblastoma’, ‘squamous cell carcinoma of lung’) were prominently enriched in genes from Q1, the regulons exhibiting the most increased variation in *DEL* compared to *CON* progenitors, and perhaps this is not surprising given the well-known role of cell cycle dysregulation in cancers. ‘Agenesis of the corpus callosum’, a sensitive neurodevelopmental indicator, is similarly represented across quintiles, while DisGeNET terms associated with neurodevelopmental disorders (‘Developmental delay (disorder)’, ‘Neurodevelopmental disorders’) were most significantly enriched in Q5, representing regulons with decreased variation in *DEL* compared to *CON* progenitors. Together, this suggests that abnormal gene expression variation correlates with phenotypic outcomes.

### Increased gene-set expression variability in DEL progenitors extends to randomly generated gene-sets

**Figure 4:**
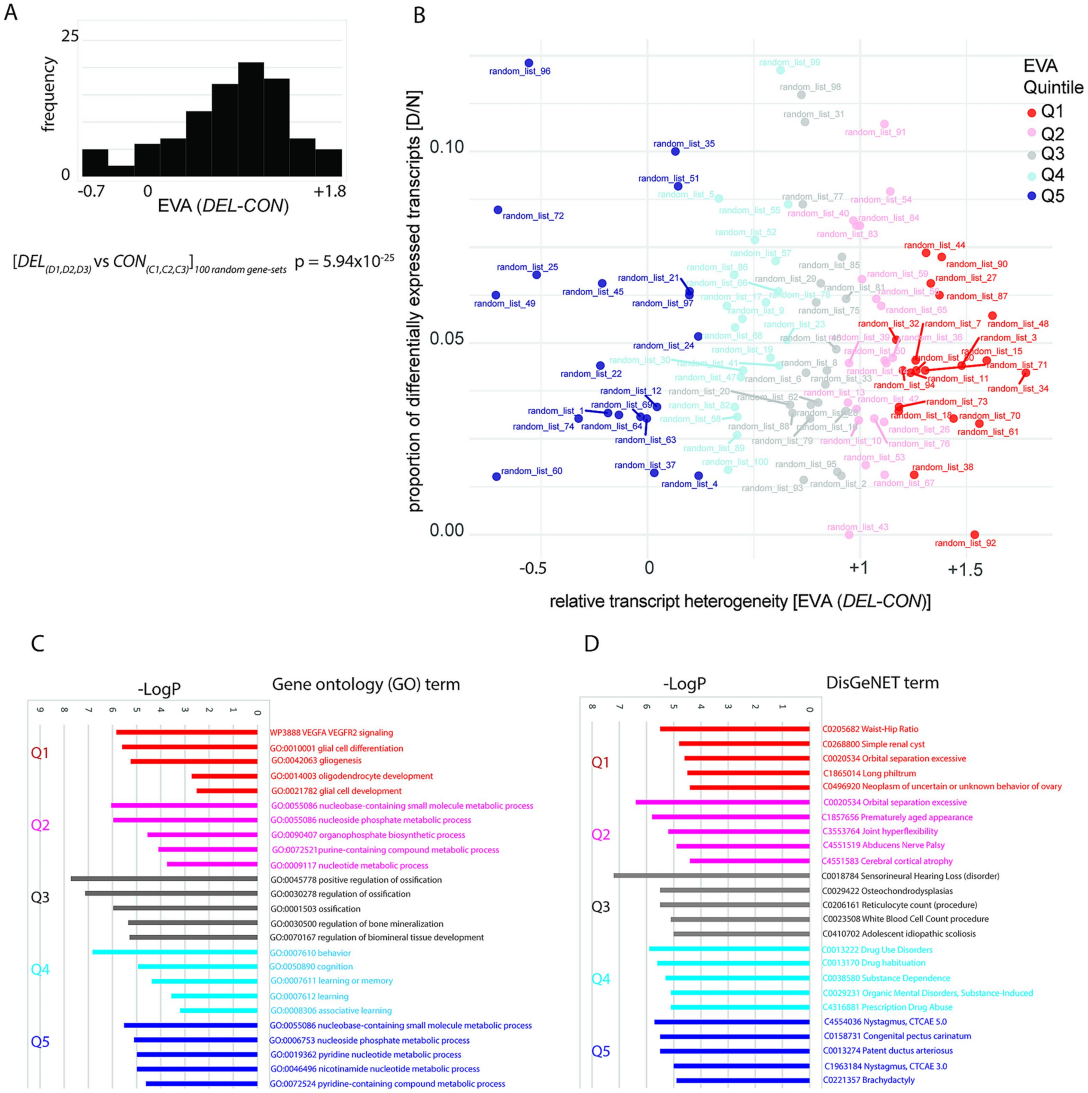
**(A,B)** Comparative expression variation analysis (EVA) of 100 random gene sets in CON and DEL progenitor cell populations. EVA(DEL-CON) is the difference between the average EVA scores of all DEL and CON progenitor samples, with a positive score indicating increased gene expression variability in DEL samples. **(A)** The distribution of EVA(DEL-CON) values shows a bias to positive scores. Paired t-test comparing EVA values for each random gene-set between CON and DEL samples (*n* = 100 gene-sets, 3 samples per genotype) *p* = 5.94×10-25. **(B)** Relationship between the EVA(DEL-CON) value for each random gene-set and the proportion of transcripts in that gene-set upregulated or downregulated more than 2 fold (D/N). The random gen-set is indicated. Q1-Q5 indicate the quintiles used for gene ontology and DisGeNET, with Q1 comprising random gene-sets with the greatest increased relative variability in DEL samples through to Q5 with the least relative variability. **(C)** Gene ontology (GO) and **(D)** DisGeNET analysis of the combined random genes in each of the quintiles. In **(C,D)**, the top 5 enriched terms for each quintile and their –log10P values are indicated.

To investigate whether the increased regulon gene expression variation in *DEL* progenitors applied more generally, we generated 100 random gene sets each comprising 100 genes (Supplementary Table S8) and subjected them to exactly the same EVA analysis described above for the regulon gene sets (Supplementary Table S9). A single omnibus paired t-test across all random gene-sets, paired by gene-set, comparing the EVA scores in *DEL* vs. *CON* samples revealed a highly significant global difference in EVA values between *CON* and *DEL* progenitors (*n* = 94 regulons, 3 sample of each genotype paired t-test *p* = 5.94×10^-25^) with EVA(*DEL-CON*) values showing a strong bias toward positive values (Figure 4A) indicating increased global gene expression variability in *DEL* compared to *CON* progenitor populations even in unrelated sets of genes. There was a trend for greater relative gene expression heterogeneity in the random gene-sets than the regulons (compare Figure 3A to Figure 4A), indicating that regulons are inherently more resistant to destabilization by the *16p11.2* microdeletion. There was a weak negative correlation (correlation coefficient = -0.13) between relative gene expression variation (EVA(*DEL-CON*)) and the proportion of differentially expressed genes (D/N) for the random gene-sets (Figure 4B). We next ranked the 100 random gene sets according to EVA(*DEL-CON*) values and divided them into quintiles of 20 gene sets each to assess enrichment of GO and DisGeNET terms. In contrast to the regulons, and not particularly surprisingly given the gene-sets were randomly generated with no functional connection, enrichment was much more modest and less obviously linked to relevant processes (Figure 4C) or diseases (Figure 4D) than observed for the regulons (Figures 4C,D), also providing a control for the substantial enrichment we observed in equivalent analysis of the regulons.

## Discussion

The *16p11.2* microdeletion is associated with the symptoms of *16p11.2* syndrome with considerable variation between individual carriers. In the current study, we investigate the transcriptional consequences of the *16p11.2* microdeletion for ventral telencephalic IN progenitor cells toward the end of the first trimester. We differentiated IPSC cells using a ventralizing protocol, including the SHH agonist purmorphamine, reported to generate GABAergic forebrain interneurons in 2D culture (Liu et al., 2013). So far as we were able to tell, examining a combination of individual marker transcripts and global comparative transcriptomics, our cultures predominantly produced a mixture of GABAergic IN progenitors and precursors resembling those found in the LGE, MGE, and CGE of the fetal ventral telencephalon *in vivo,* with little evidence of dorsal telencephalic character. However, and this applies generally to culture-derived IPSC cell types, we are mindful that our differentiation conditions are extremely unlikely to perfectly recapitulate physiological conditions, and our cells may also have broader neural progenitor character. This does not affect our main conclusions about the effect of the *16p11.2* microdeletion on otherwise isogenic progenitor cells cultured under equivalent conditions.

Examination transcripts with significantly different average transcript levels (DEGs) between *CON* and *DEL* progenitors revealed that even at the IN progenitor stage, enrichment of disease genes (DisGeNET) associated with epilepsy, a condition found in 20% of *16p11.2* microdeletion carriers, dominated, while prominent among gene ontology (GO) terms enriched in the DEGs were calcium signaling and the synapse. This aligns with studies where IPSC cells with and without the *16p11.2* microdeletion are differentiated into neural progenitors, revealing hundreds of relatively modestly upregulated or downregulated DEGs, with a common theme of involvement in cell signaling and neurodevelopmental disorders (Blumenthal et al., 2014; Connacher et al., 2022; Roth et al., 2020; Urresti et al., 2021). We also employed approaches relying on transcript-level fold change values between *CON* and *DEL* progenitors regardless of the statistical significance of individual DEGs. GSEA found enriched terms linked to cancer and histone modification. A comparative transcriptomic proxy of polygenic risk scores calculated for autism and obesity by combining fold-change transcript downregulation in *DEL* samples with GWAS data indicated increased risk for both autism and obesity in DEL.

In a recent study, we differentiated isogenic human *CON* (*16p11.2^+/+^*) and *DEL* (*16p11.2^+/-^*) IPSC cells into ventral telencephalic organoids and found that the *DEL* organoids exhibited a phenotype of increased variability in organoid size, cell cycle kinetics, and accelerated maturation (Fetit et al., 2023). This suggested that increased phenotypic variability in organoid culture is a consequence of reduced dosage of the *16p11.2* locus itself. This all prompted us to ask whether focusing on gene expression variability rather than solely on differential average expression would give us additional insight into the *16p11.2* phenotype, and we used single-cell expression variation analysis (EVA) to compare variation in the expression of gene-sets between individual cells in the *CON* and *DEL* progenitor cell populations (Davis-Marcisak et al., 2019). We examined 94 regulons identified by SCENIC in our single-cell transcriptomes and found a strong bias toward increased gene expression variation in samples harboring the *16p11.2* microdeletion. The most variable 19 regulons (Quintile 1) presented the strongest enrichment in GO terms linked to the cell cycle and DisGeNET terms associated with cancer. The significant overlap between genes associated with cancer and those associated with autism and other neurodevelopmental conditions has been interpreted as indicating common underlying molecular mechanisms (Forés-Martos et al., 2019). Directly relevant to the current study, the *16p11.2* microdeletion increases the odds of developing neuroblastoma 13-fold, comparable to increased odds for other *16p11.2* symptoms, revealing dual function in cancer and neurodevelopment (Egolf et al., 2019). While we previously found that ventral telencephalic organoids harboring the *16p11.2* microdeletion generally presented more variable phenotypes than their wild-type counterparts, this study did not address cell–cell variation (Fetit et al., 2023). Our finding here that the *16p11.2* microdeletion increases gene expression heterogeneity between cells is reminiscent of cancers, where EVA analysis of single cell transcriptomes reveals increased gene expression variation in large numbers of tumor samples compared to their non-tumorous counterparts (Davis-Marcisak et al., 2019). Increased cell–cell variability in regulon transcript levels associated with the cell-cycle extends our earlier finding of increased organoid–organoid cell-cycle variability (Fetit et al., 2023). Less expected was that this effect was much more widely distributed across the genome, as randomly selected gene sets also showed a strong bias to increased gene expression variation between cells harboring the *16p11.2* microdeletion. Together, this prompts the hypothesis that tumors and neurodevelopmental conditions share overlapping gene expression heterogeneity as an underpinning phenotype.

Our finding that the dosage of the *16p11.2* locus has a widespread influence on cell–cell transcriptional variation for thousands of genes scattered about the human genome poses the question of the impact. Given the highly constrained boundaries for cell function, it is easy to imagine that global transcript instability, with knock-on consequences for biomolecule interactions, has far-reaching consequences. In addition to cancer biology, where molecular heterogeneity has long been associated with disease progression, it has more recently been reported that gene expression variation associates with other neurodevelopmental conditions. An elegant recent study finds increased gene expression variability in human IPSC-derived neurons harboring trisomy 21 or *CHD8* mutations, both risk factors for neurodevelopmental conditions (Upadhya et al., 2025). Trisomy 21 is a polygenic risk factor increasing the dosage of hundreds of genes on chromosome 21, and it is unknown which of these genes underlie the gene expression variation phenotype. The *CHD8* mutation is a monogenic risk factor, and *CHD8* mutations likely have an impact on gene expression variability stemming from their role in chromatin remodeling (Wang et al., 2017). GSEA analysis (current study) identified enrichment and depletion of transcripts associated with chromatin biology in *DEL* progenitors, consistent with the idea that chromatin disruption is a factor in the *16p11.2* phenotype. The *16p11.2* locus includes *HIRIP3* and *PAGR1,* which both function in chromatin biology, suggesting the hypothesis that their reduced dosage caused by the *16p11.2* microdeletion impacts gene expression variation by disrupting chromatin. It will also be of interest to establish whether the phenomena of increased gene expression variability extend to other neurodevelopmental disorder risk factors in addition to *CHD8*, trisomy 21, and *16p11.2*.

One likely consequence of increased transcript expression variability within regulons is disruption of the processes normally controlled by that regulon. Indeed, several of the most disorganized regulons in Quintile 1 (*THRA(+)*, *RFX4(+)*, *POU3F2(+)*, *PAX6(+)*, *E2F1(+)*, and *NR2F1(+)*) are regulated by TFs (the hub TFs) associated with *16p11.2* symptoms. Thyroid hormone (TH) acts by binding to its receptor encoded by *THRA,* enabling it to activate gene expression by binding to thyroid response elements (TRE) in target gene promoters, and dysregulation of thyroid signaling is associated with a wide variety of symptoms, with *THRA* mutations identified as risk factors for obesity and autism (Fernández-Real et al., 2013; Kalikiri et al., 2017; Yuen et al., 2015). Obesity has also been associated with altered chromatin accessibility of TRE sites to THRA protein in adipocytes, suggesting that *THRA* phenotypes can be mediated by dysregulating THRA-activated transcription without directly affecting THRA itself (Zhu et al., 2025). Cilia are cellular antennas essential for many signaling pathways, and mutations affecting cilia components predispose to symptoms overlapping with *16p11.2* syndrome, including obesity and autism, with disruption to cilia reported in cells harboring the *16p11.2* microdeletion (Harris et al., 2021; Migliavacca et al., 2015; Schwartz et al., 2011). *RFX4*, along with several other *RFX* transcription factors, regulates transcription of cilia genes, and RFX4 binds to promoters and regulates *POU3F2* and *NEUROD2* proneural gene transcription in human neural progenitors (Choi et al., 2024). Notably, *POU3F2* loss-of-function alleles associate with obesity and neurodevelopmental disorders (Schönauer et al., 2023). *PAX6* mutations were identified decades ago as risk factors for the ocular condition aniridia, and more recently, there is accumulating evidence that heterozygosity for *PAX6* loss of function also affects brain anatomy and function (Abouzeid et al., 2009; Bamiou et al., n.d.; Grant et al., 2021; Hanish et al., 2016; Maekawa et al., 2009; Manuel et al., 2015; Yogarajah et al., 2016). Elevated *E2F1* levels have been associated with obesity in humans, and there is evidence for *E2F1* acting both in adipose cells and in the hypothalamic appetite-regulating cells to regulate metabolism and appetite (Denechaud et al., 2017; Haim et al., 2015; Lu et al., 2013). *NR2F1* is expressed in IN progenitors and associated with autism (Zhang et al., 2020). Overall, the approach of using variation as a criterion over and above a sole focus on differential average gene expression has yielded regulons with known clinical impact that merit further investigation. Given the widespread expression of the *16p11.2* locus, it will be interesting to see whether this enhanced molecular variability phenotype of the *16p11.2* locus applies more broadly and investigate the mechanism and phenotypic consequences in more depth.

## Data Availability

Single-cell RNA sequencing data is available via European Nucleotide Archive (ENA) accession code PRJEB46758. https://www.ebi.ac.uk/ena/browser/view/PRJEB46758. Code is available on GitHub https://github.com/16p11-iPSC/16p11.2-iPSC-scRNAseq.git.

## Footnotes

1
http://metascape.org

2
https://molbiotools.com/

## Supplementary material

SUPPLEMENTARY FIGURE S1 SNP microarray: Single nucleotide polymorphism (SNP) analysis of IPSC lines showing genomic copy number variation. GM8 is the ancestral line used to derive the control [FACS52 (C1), FACS53 (C2 and C3)] and *16p11.2* microdeletion [DELD5 (D1), DELH7 (D3), and DELC5 (D2)] samples as described previously (Tai et al., 2016). The key indicates different types of copy number variation and the position of the 16p11.2 locus on chromosome 16 is indicated confirming a heterozygous deletion in DEL and not CON lines. No other copy number variation consistently partitions between *CON* and *DEL* lines confirming they are overwhelmingly isogenic except for the *16p11.2* locus itself.

SUPPLEMENTARY FIGURE S2 IPSC culture: **(A)** Summary of culture protocol used to differentiate *CON* and *DEL* isogenic IPSC cells (Liu et al., 2013) for 80 days prior to dissociation for single cell RNA sequencing. **(B–D)** Immunofluorescence for FOXG1 and TUJ1 on a *CON* **(B, C)** and *DEL* **(D, E)** culture after 80 days in culture. **B, D** shows the FOXG1 signal only while **C, E** show the TUJ1, FOXG1 and DAPI channels merged.

SUPPLEMENTARY TABLE S1 DESeq2 DEGs: DESeq2 output showing differential transcript levels between *CON* and *DEL* progenitors samples. First column indicates transcript with information about each transcript shown in the corresponding row. Columns: ‘pct_exp_CON_Prog’ and ‘pct_exp_DEL_Prog’ show the percentage of cells expressing each transcript in *CON* and *DEL* progenitor samples respectively; ‘baseMean_Prog’ shows overall transcript expression level; ‘Log2FoldChange’ is the fold change in *CON* relative to *DEL* progenitors as a Log to the base 2 with negative values indicating reduced expression in *DEL* compared to *CON* (for example Log2FC = -1.0 indicates 2-fold down regulation in *DEL* compared to *CON*). ‘LfcSE’ is the standard error for the Log2FoldChange; and ‘pvalue’ and ‘padj’ are the unadjusted and adjusted *p*-values associated with differential expression.

SUPPLEMENTARY TABLE S2 Autism BETA: First column indicates gene with information about each shown in the corresponding row. Columns: ‘autism_OR’ is the odds ratio for autism associated with each gene; and ‘ASD_BETA’ the effect size for association with autism (see Methods) calculated as BETA = base_e_autism_OR.

SUPPLEMENTARY TABLE S3 BMI BETA: First column indicates gene with information about each shown in the corresponding row. Columns: ‘BMI_mean_BETA’ is the effect size for association with BMI (Obesity) calculated by averaging the individual BETA values of SNPs in a 32.5 kilobase pair window encompassing each gene (see Methods).

SUPPLEMENTARY TABLE S4 SCENIC regulons DEL: First column indicates the HubTF for each regulon and the second column indicates it’s transcriptional targets as identified by SCENIC in the *DEL* single cell transcriptomes (see Methods).

SUPPLEMENTARY TABLE S5 SCENIC regulons CON: First column indicates the HubTF for each regulon and the second column indicates its transcriptional targets as identified by SCENIC in the *CON* single cell transcriptomes (see Methods).

SUPPLEMENTARY TABLE S6 EVA for regulons *DEL*: First column indicates the HubTF for each regulon identified by SCENIC in *DEL* samples with information about each shown in the corresponding row. Columns: ‘N_Prog’ is the number of expressed transcripts in each regulon; ‘D_Prog’ is the number of transcripts expressed more than 2-fold differently between *CON* and *DEL* samples; ‘D-N_Prog’ is D divided by N (D/N - the proportion of transcripts in each regulon expressed more than 2-fold differently between *CON* and *DEL* samples); The remaining columns list the EVA values for each *CON* (‘Prog_C1’, ‘Prog_C2’, and ‘Prog_C3’) and *DEL* (‘Prog_D1’, ‘Prog_D2’, and ‘Prog_D3’) progenitor sample (see Methods).

SUPPLEMENTARY TABLE S7 EVA for regulons *CON*: First column indicates the HubTF for each regulon identified by SCENIC in *CON* samples with information about each shown in the corresponding row. Columns: ‘N_Prog’ is the number of expressed transcripts in each regulon; ‘D_Prog’ is the number of transcripts expressed more than 2-fold differently between *CON* and *DEL* samples; ‘D-N_Prog’ is D divided by N (D/N - the proportion of transcripts in each regulon expressed more than 2-fold differently between *CON* and *DEL* samples); The remaining columns list the EVA values for each *CON* (‘Prog_C1’, ‘Prog_C2’, and ‘Prog_C3’) and *DEL* (‘Prog_D1’, ‘Prog_D2’, and ‘Prog_D3’) progenitor sample (see Methods).

SUPPLEMENTARY TABLE S8 Random gene sets: Each row shows a list of 100 randomly selected genes. The first column shows the identifier used to refer to each gene set.

SUPPLEMENTARY TABLE S9 EVA random gene sets: First column indicates the arbitrary identifier for each randomly generated gene set of 100 genes with information about each shown in the corresponding row. Columns: ‘D-N_Prog’ is the proportion of transcripts in each rgene-set expressed more than 2-fold differently between *CON* and *DEL* samples; The remaining columns list the EVA values for each *CON* (‘Prog_C1’, ‘Prog_C2’, and ‘Prog_C3’) and *DEL* (‘Prog_D1’, ‘Prog_D2’, and ‘Prog_D3’) progenitor sample (see Methods).
