# Supplementary figures and images for: 16p11.2 microdeletion enhances gene expression variability between human IPSC-derived forebrain interneuron progenitor cells in culture
Source: Front Mol Neurosci. 2026 Jul 8;19:1872405. doi: 10.3389/fnmol.2026.1872405 (PMC13388902; doi:10.3389/fnmol.2026.1872405)

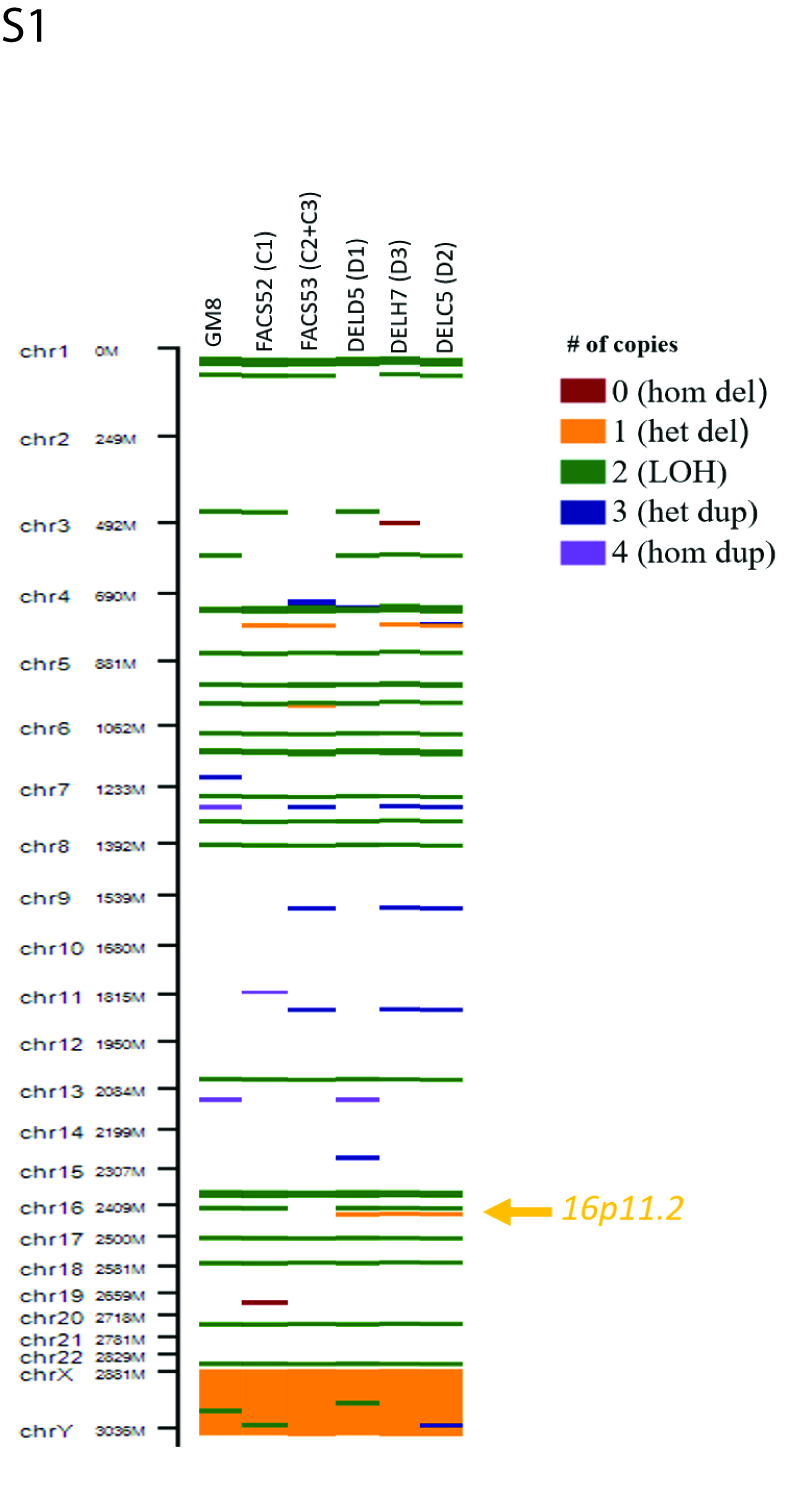

Supplement: SUPPLEMENTARY FIGURE S1 — SNP microarray: Single nucleotide polymorphism (SNP) analysis of IPSC lines showing genomic copy number variation. GM8 is the ancestral line used to derive the control [FACS52 (C1), FACS53 (C2 and C3)] and 16p11.2 microdeletion [DELD5 (D1), DELH7 (D3), and DELC5 (D2)] samples as described previously (Tai et al., 2016). The key indicates different types of copy number variation and the position of the 16p11.2 locus on chromosome 16 is indicated confirming a heterozygous deletion in DEL and not CON lines. No other copy number variation consistently partitions between CON and DEL lines confirming they are overwhelmingly isogenic except for the 16p11.2 locus itself. [file Image_1.TIF]

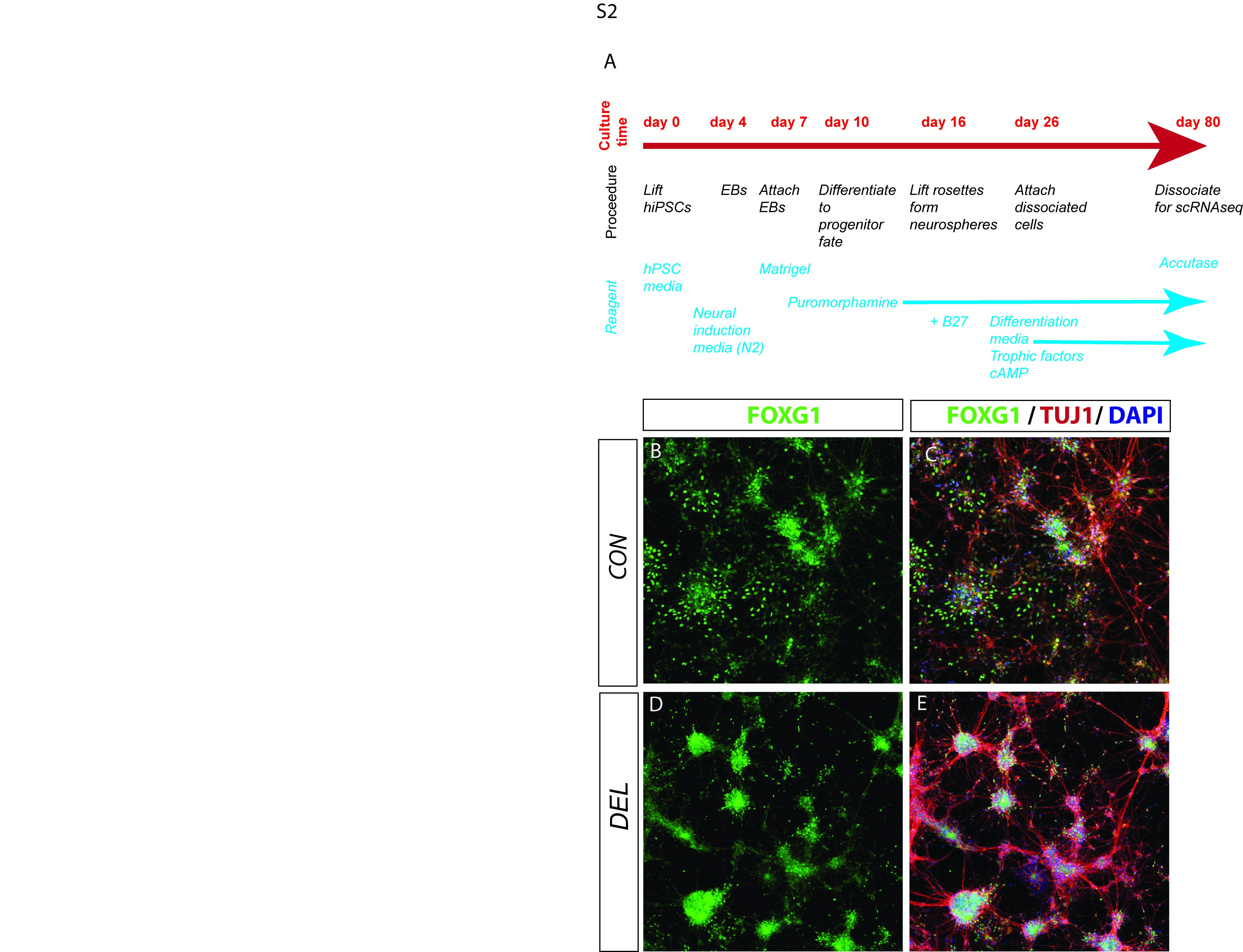

Supplement: SUPPLEMENTARY FIGURE S2 — IPSC culture: (A) Summary of culture protocol used to differentiate CON and DEL isogenic IPSC cells (Liu et al., 2013) for 80 days prior to dissociation for single cell RNA sequencing. (B–D) Immunofluorescence for FOXG1 and TUJ1 on a CON (B, C) and DEL (D, E) culture after 80 days in culture. B, D shows the FOXG1 signal only while C, E show the TUJ1, FOXG1 and DAPI channels merged. [file Image_2.TIF]
